# Understanding the implementation of evidence-informed policies and practices from a policy perspective: a critical interpretive synthesis

**DOI:** 10.1186/s13012-021-01082-7

**Published:** 2021-02-15

**Authors:** Heather L. Bullock, John N. Lavis, Michael G. Wilson, Gillian Mulvale, Ashleigh Miatello

**Affiliations:** 1 Department of Health Research Methods, Evidence and Impact, McMaster University, 1280 Main Street West, Hamilton, Ontario L8S 4L6 Canada; 2 McMaster Health Forum, Hamilton, Canada; 3 DeGroote School of Business, McMaster University, Burlington, Canada

**Keywords:** Implementation science, Public policy, Evidence-based health care, Systematic review, Critical interpretive synthesis

## Abstract

**Background:**

The fields of implementation science and knowledge translation have evolved somewhat independently from the field of policy implementation research, despite calls for better integration. As a result, implementation theory and empirical work do not often reflect the implementation experience from a policy lens nor benefit from the scholarship in all three fields. This means policymakers, researchers, and practitioners may find it challenging to draw from theory that adequately reflects their implementation efforts.

**Methods:**

We developed an integrated theoretical framework of the implementation process from a policy perspective by combining findings from these fields using the critical interpretive synthesis method. We began with the compass question: How is policy currently described in implementation theory and processes and what aspects of policy are important for implementation success? We then searched 12 databases as well as gray literature and supplemented these documents with other sources to fill conceptual gaps. Using a grounded and interpretive approach to analysis, we built the framework constructs, drawing largely from the theoretical literature and then tested and refined the framework using empirical literature.

**Results:**

A total of 11,434 documents were retrieved and assessed for eligibility and 35 additional documents were identified through other sources. Eighty-six unique documents were ultimately included in the analysis. Our findings indicate that policy is described as (1) the context, (2) a focusing lens, (3) the innovation itself, (4) a lever of influence, (5) an enabler/facilitator or barrier, or (6) an outcome. Policy actors were also identified as important participants or leaders of implementation. Our analysis led to the development of a two-part conceptual framework, including process and determinant components.

**Conclusions:**

This framework begins to bridge the divide between disciplines and provides a new perspective about implementation processes at the systems level. It offers researchers, policymakers, and implementers a new way of thinking about implementation that better integrates policy considerations and can be used for planning or evaluating implementation efforts.

Contributions to the literature

- This study unpacks the implementation of evidence-informed policies and practices through the systematic development of new theory drawing from three distinct fields of scholarship: policy implementation, implementation science, and knowledge translation, answering a call from implementation researchers for more integration.
- The conceptual framework views implementation from the “outer context” and includes (1) a model describing the process of implementation and (2) a framework that identifies the policy-related determinants of implementation success.
- This conceptual framework provides researchers, policymakers, and implementers with a new way of thinking about implementation and can be used for planning or evaluating implementation efforts.

## Background

Implementation has captured the attention of public policy scholars for well over 50 years [1], yet remains relatively under-studied compared to other stages of policy-making. The reasons for this are many and include challenges with isolating implementation from other parts of the policy process and a lack of agreement about conceptual underpinnings [2]. This then leads to challenges in identifying relevant explanatory variables and analysts often must resort to a “long list of variables that are potentially useful” [2]. Even once decisions regarding these challenges have been made, the complex, multi-level, and multi-faceted nature of implementation creates difficulties designing and conducting high-quality empirical research that can offer useful generalizations to those interested in improving the process of implementation and thus achieving better policy results [2].

Research on implementation has also independently come into sharp focus through the related fields of knowledge translation and implementation science. Conceptual work on implementation from these fields has increased at a seemingly exponential rate to the point where there is a great deal of focus on sorting and classifying the many frameworks, models, and theories and providing guidance toward their use [3–6]. The empirical literature is also rapidly increasing, with over 6200 systematic reviews on consumer-targeted, provider-targeted, and organization-targeted implementation strategies in the health field alone (based on a search of www.healthsystemsevidence.org).

Despite the large number of models, theories, and frameworks being generated in the knowledge translation and implementation science fields, the role of policy in the implementation process appears to be under-theorized. When policy is included in conceptual work, it is often identified as a contextual variable [7, 8] rather than being central to the implementation concept itself. It is also often presented as a broad category of “policy”, rather than as a variable that is specific and therefore measurable in empirical work. This lack of conceptual clarity and empirical work about policy and other policy-related structural constructs has been noted by several researchers. For example, a systematic review of measures assessing the constructs affecting implementation of health innovations makes specific reference to the “relatively few” measures available to assess structural constructs, which they define as “political norms, policies and relative resources/socio-economic status” [9]. As a result, the field of public policy appears to have on the one hand, a challenge of too many policy-related implementation variables, and on the other hand, the fields of knowledge translation and implementation appear to have too few.

In recent years some researchers have recognized these silos in scholarship and have called for more implementation research that integrates public policy and implementation science and knowledge translation perspectives [10]. For example, Johansson concludes that implementation problems could be better understood through the inclusion of research in public administration, with more focus on issues such as resource allocation, priorities, ethical considerations, and the distribution of power between actors and organizational boundaries [11].

In addition to these challenges, much of the seminal policy scholarship on implementation from both the public policy and knowledge translation and implementation literatures come from the USA [12–15]. This has resulted in a concentration of theoretical and empirical works that reflect the governance, financial and delivery arrangements that are particular to the USA [16, 17] and that may not always readily apply in other contexts. These differences are particularly marked when it comes to the policy domain of health given the differences of the US system compared to most others [18]. One notable exception to this is the European contributions to the “second generation” of policy scholarship on implementation, which adopted the perspective of those at the “coal face” of policy implementation [19].

In response to these challenges, the objective of our study was to develop an integrated theoretical framework of the implementation process from a policy perspective by combining findings from the public policy, implementation science, and knowledge translation fields. By integrating knowledge from these fields using a critical interpretive synthesis approach, we specifically examine how policy considerations are described in implementation theories, frameworks, and processes from existing published and gray literature. Our goal was to generate a theoretical framework to foster an improved understanding of the policy contributions to implementation that can be used in future studies to generate testable hypotheses about large-scale system implementation efforts.

## Methods

### Study design

Given the broad goal of this study, the question of interest, and the scope of potentially applicable literature from discrete fields that could inform this work, we selected a critical interpretive synthesis (CIS) approach. Drawing from the techniques of meta-ethnography combined with traditional systematic review processes, CIS employs an inductive and interpretive technique to critically inspect the literature and develop a new conceptualization of the phenomenon of interest. Unlike traditional systematic reviews that often focus on questions of effectiveness, CIS is helpful in generating mid-range theories with strong explanatory power [20, 21]. This is suitable for our goal of developing a conceptual framework that better integrates findings from diverse fields and affords the opportunity to critically inspect both individual studies and the literature from each field as a whole in terms of the nature of the assumptions underlying each field, and what has influenced their proposed solution [22]. The method begins with a compass question, which evolves throughout the course of the review [22, 23]. Our compass question was as follows: How is policy currently described in implementation theory and processes and what aspects of policy are important for implementation success?

### Review scope

Our review casts a very broad net in terms of implementation processes and theories. While our main focus is on large-scale implementation efforts in health, behavioral health, and human services areas that are not specific to a particular condition, we also drew from other large-scale implementation theories and empirical work, such as from the field of environmental science, that may yield important insights toward a more integrated framework of implementation. We drew from two key sources of literature: (1) existing frameworks, models, and theories (public policy, implementation science and knowledge translation) and (2) empirical studies that report on specific implementation processes.

Given our interest in implementation processes from a policy perspective, we limited our review to implementation frameworks, models, theories and empirical reports that describe implementation efforts at a community or systems level (e.g., city, province/state or country) where policy considerations are most likely to be an important factor. Implementation of a single evidence-based practice (unless across a large-scale) or implementation in a single organization were excluded, as was research that focused on behavior change at the individual level.

### Electronic search strategy

Using the compass question, and in consultation with a librarian, we constructed a table of Boolean-linked key words and then tested several search strategies (Table 1). The search was then conducted in October 2020 for the time period of January 2000–September 2020 using the following 12 databases: ASSIA, CINAHL (via EBSCO), EMBASE (via Ovid), ERIC, Health Star (via Ovid), MEDLINE (via Ovid), PAIS Index, PolSci, PsychINFO, Social Sciences Abstracts, Social Services Abstracts, and Web of Science. The dates for the policy databases (PolSci and Social Sciences Abstracts) were extended to 1973 to ensure key conceptual articles would be retrieved, such as the seminal work by Sabatier and Mazmanian in 1980 [14]. A gray literature search was also conducted using Health Systems Evidence (which indexes policy documents related to health system arrangements and implementation strategies, as well as systematic reviews). Similar search strings were used across all databases with minor adjustments to ensure searches were optimized. We prioritized sensitivity (comprehensiveness) over specificity (precision) in our search strategy.

**Table 1:** Search terms

Implementation terms		Government level		Organizational level		Practice level		Evidence terms (with and without dashes)
implement*	**AND**	policy	**OR**	organizational polic*	**OR**	“clinical guideline”	**AND**	“evidence-based practice*”
“knowledge translation”		strategy		policy and procedures manual		“practice guideline”		“evidence-informed practice*”
“knowledge mobili*”		“action plan”		procedures manual		scope of practice		“evidence-informed policy”
								“evidence-based policy”

#### Article selection

We excluded articles based on their titles and abstracts if they did not fit within the study scope or if they were not conceptual or empirical works. We created additional inclusion criteria that were based on the following questions: (1) Is there a moderate (or greater) chance that the article will shed light on the role of policy in an implementation process or on the outcomes of the process? (2) Does the article describe implementation efforts at a community or systems level? And (3) does the article identify actors at the government, organizational or practice level such as policy entrepreneurs who may be central to policy implementation efforts? Any articles that did not meet at least one of these criteria were excluded.

Complementary to the formal search and in keeping with the inductive strategies that are part of the CIS process, we also conducted hand searches of the reference lists of relevant publications and searched the authors’ personal files to identify further articles and theoretically sampled additional articles to fill conceptual gaps as the analysis proceeded.

After completing the searches, an Endnote database was created to store and manage results. Once duplicates were removed, a random selection of two percent of the articles was independently screened by two reviewers (H.B. and A.M.) who were blinded to each other’s ratings and used the same inclusion criteria. The reviewers classified each title and abstract as “include”, “exclude”, or “uncertain”. Inter-rater agreement was determined using the kappa statistic. This process was undertaken to improve the methodological rigor by enhancing trustworthiness and stimulating reflexivity, not to establish a quantitative assessment per se [24]. Any discrepancies were then discussed between reviewers until consensus was reached. Next, one reviewer assessed the remaining titles and abstracts. Articles classified as “include” or “uncertain” were kept for full text review.

The full text of the remaining articles was then assessed by one reviewer. Articles were excluded at this stage if they did not provide detailed insight into the compass question. Articles were also sorted according to whether they were a conceptual contribution (i.e., presented a model, theory, framework or theoretical concept on implementation) or an empirical contribution (i.e., used qualitative, quantitative, review or other research methods to present new findings, or an analysis of implementation).

### Data analysis and synthesis

Our data analysis proceeded in four stages. First, while screening and assessing the articles for inclusion, we noted some general observations of how policy was incorporated in the literature from each field of interest (policy/public administration, implementation, and knowledge translation). Second, we classified articles according to how policy was portrayed in implementation theory and processes. Third, we constructed a data extraction template for conceptual and empirical studies that included (1) descriptive categories (the author(s), the name of the model, theory or framework (if provided), year of publication, author location, focus of the article, and whether a graphic or visual aid was included), (2) content from the article that addressed the compass question regarding how policy is portrayed and what aspects are important for success, and (3) interpretive categories including “synthetic constructs” developed by the review team from the article and additional notes on how the article contributed to the development of the conceptual model. Additionally, the data extraction form for the conceptual articles included a classification of the type of framework according to Nilsen’s taxonomy of implementation models, theories, and frameworks [3].

In the fourth and final stage, we initially focused on the conceptual literature and used it as a base from which to build our integrated conceptual model. We developed the synthetic constructs by reviewing the content from each article that addressed the compass question and interpreting the underlying evidence using a constant comparative method to ensure that the emerging synthetic constructs were grounded in the data, similar to a grounded theory approach [25]. These synthetic constructs were then used to begin to build the conceptual model and an accompanying graphic representation of it. We then critiqued the emerging constructs to identify gaps in the evidence and emerging constructs.

Using this emerging model, we purposively sampled additional conceptual literature to fill the gaps that we identified and to ensure we incorporated as many relevant concepts as possible. We did this by consulting reviews of existing models, theories, and frameworks [2–6] to identify additional relevant concepts not captured by our search strategy and by hand searching the reference sections of some seminal conceptual papers [7, 26]. Once saturation of the conceptual literature was reached, we purposively sampled a subset of the empirical literature and used this subset to “test” the model and add additional detail to the theoretical constructs gleaned from empirical report. We used a similar data extraction template with the exceptions of removing the descriptive category of model or theory name and the interpretive classification using the Nilsen taxonomy [3], but adding the descriptive category of “methodology”. If our model did not capture findings from the empirical studies, we revised it and re-tested. This process continued until saturation was reached and additional empirical studies yielded no further insights into our model.

The methods reported here are based on a protocol developed prior to initiating the study. The protocol and a note about the four ways that the reported methods differed from the protocol are available upon request.

## Results

### Search results and article selection

**Fig. 1:**
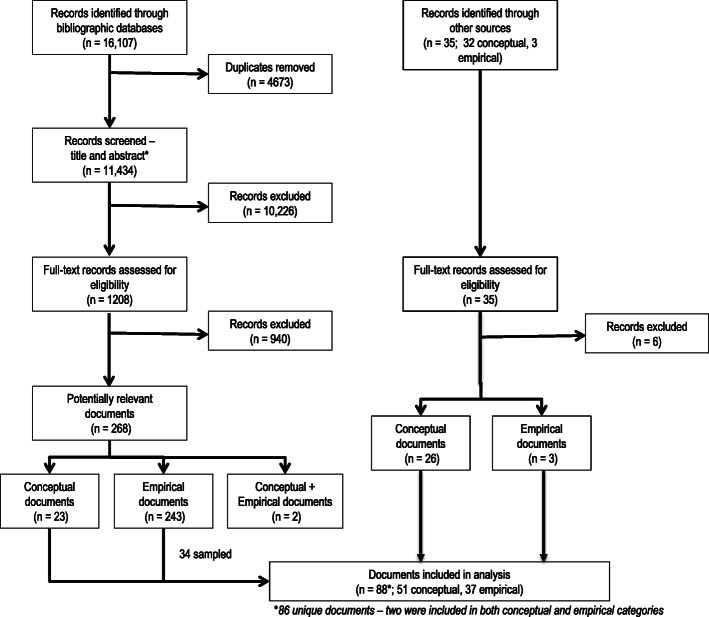
Literature search and study selection flow diagram

Our database search retrieved 16,107 documents and 11,434 unique documents once duplicates were removed. The review of titles and abstracts was completed independently by two reviewers on a random sample (*n* = 171) of the documents. The Kappa score was 0.72 indicating substantial agreement. Figure 1 provides a flow diagram outlining the search strategy. Following these criteria for the remaining titles and abstracts resulted in 1208 documents included for full text review. The full text review excluded an additional 940 documents leaving 268 potentially relevant documents (excluded documents and the rationale for exclusion are available upon request). Of these, 23 conceptual documents, 243 empirical documents, and two documents that included both conceptual and empirical elements were included for the data extraction and analysis phase. We sampled and extracted data on all of the conceptual articles. For the empirical articles, we chose a maximum variation sampling approach based on the subject matter and article topic with an initial sample of 10% of the articles. We also noted that nine of the articles related to a large, multi-year national implementation study [27–35]. Because this was the largest and most comprehensive account of the role of policy in large-scale implementation efforts identified through our search, we included these as a sub-group for data extraction. This approach led to data extraction for 34 empirical articles.

In addition to these two approaches, we sampled articles that filled in conceptual gaps as our model developed. This process resulted in the retrieval of an additional 26 conceptual articles and 3 empirical articles. In total, 86 unique documents were included with two of these documents used in both the conceptual and empirical data extraction (Tables 2 and 3). While our process was inclusive of English language publications from any country, the majority of articles were conducted by US researchers (*n* = 57), with the others coming mainly from other Western countries (the UK (*n* = 8), the Netherlands (*n* = 7), Australia (*n* = 5), Canada (*n* = 2), Sweden (*n* = 2), Germany (*n* = 2), and Europe, China, and OECD (*n* = 1)). Articles covered a range of topics including health and health care, public health, mental health and addictions, children and youth, social care, justice, and climate change, among others. The conceptual documents included all of the categories of theories, models and frameworks identified by Nilsen [3], with the Determinants Framework type being most common. The empirical articles employed a wide array of methods that fall into the broad categories of qualitative, quantitative, and mixed methods.

**Table 2:** Overview of included conceptual literature

Author	Year	Author location	Topic area	Focus	Name of model/theory/framework	Framework type [3]
Aarons et al. [12]	2011	USA	Public services for children and families	Implementation	Conceptual model of global factors affecting implementation in public service sectors	Determinants framework
Bauman et al. [36]	2006	Australia (majority)	Physical activity	Supra-National	A Six-Step Framework for International Physical Activity Dissemination	Process model
Bowen et al. [37]	2010	USA	HIV	Organizational/program	Rogers-Rütten Framework	Determinants + evaluation framework
Bowen and Zwi [38]	2005	Australia	Public health	Knowledge translation	Evidence-informed Policy and Practice Pathway	Determinants framework
Bruns et al. [39]	2008	USA	Children and youth	System (state)	No name per se but addresses dimensions of state EBP implementation effort	Determinants framework
Burris et al. [40]	2012	USA	Public health	System (law)	No name per se but unified framework integrating public health law and public health systems and services	Determinants framework
Campos and Reich [41]	2019	USA	Health	Policy, politics and stakeholders	No name but identifies six different directions for different stakeholders	Process model
Chaudoir et al. [9]^a^	2013	USA	Health	System (measures of determinants)	A multi-level framework predicting implementation outcomes	Determinants framework
Cherney and Head [42]	2011	Australia	Evidence-based policy/practice	System	Components of a Support Delivery System: ‘9Cs’	Determinants framework
Chin and Goldmann [43]	2011	USA	Health	System	A Conceptual Model for Specifically Addressing Disparities 6 Key Levels of Influence	Implementation theory
Damschroder et al. [7]	2009	USA	Health	Organizational	Consolidated Framework for Advancing Implementation Research (CFIR)	Determinants framework
Domitrovich et al. [44]	2008	USA	Schools	Implementation quality	No name per se but identified as factors that can affect implementation quality: a multi-level model	Determinants framework
Evans and Davies [45], and Dolowitz and March [46]	1999 and 2000	UK and UK	Policy transfer	Policy	Policy transfer	Determinants framework
Feldstein and Glasgow [47]	2008	USA	Healthcare	Research to practice implementation	PRISM (Practical, Robust Implementation and Sustainability Model)	Process model
Fleuren et al. [48]	2014	Netherlands	Healthcare	Organizational/program	No name per se but “Framework representing the innovation process and related categories of determinants”	Determinants framework
Godfrey [49]	2011	USA	Mental health	System	Hypothesized factors that influence ACT implementation	Determinants framework
Green et al. [50]	2006	USA	Physical activity	Knowledge translation	Push-Pull Capacity Model	Process model
Greenhalgh et al. [26]	2004	UK	Healthcare	Organizational	Diffusion of Innovations in Service Organizations	Determinants framework
Greig et al. [51]	2012	UK	Healthcare	Implementation activity/practices	Activity Theory	Classic theory
Harris et al. [52]	2012	USA	Health promotion	Organizational	Health Promotion Resource Center Dissemination Framework	Process model
Harvey and Kitson [53]	2016	Australia	Health services	Implementation	Integrated Promoting Action on Research Implementation in Health Services (I-PARIHS)	Determinants framework
Hendriks et al. [54]	2013	Netherlands	Public health (childhood obesity)	Policy	Behavior Change Ball	Implementation theory
Hill and Hupe [55]	2003	UK and Netherlands	Policy implementation	Policy	No model/theory or framework but discussed ‘the multi-layer problem’	N/A
Hill and Hupe [56]	2002	UK and Netherlands	Policy implementation	Policy	N/A (book)	Determinants framework
Hodges and Ferreira [57]	2013	USA	Children and families	Policy (local)	Multilevel framework for local policy development and implementation	Determinants framework
Howlett [58]	2004	Canada	Policy implementation	Policy (instruments)	N/A	Other (most closely resembles Classic Theory)
Hupe [59]	2011	Netherlands	Policy implementation	Explaining policy implementation	Thesis of incongruent implementation	Determinants framework
Hupe and Hill [60]	2016	Netherlands and UK	Policy implementation	Policy	N/A	N/A
Jansen [61]	2010	Netherlands	Public health	Disconnections between policy, practice and research	3 niches of public health	Process model + determinants framework
Jilcott et al. [62]	2007	USA	Public health	Evaluating policy implementation	Applying the RE-AIM framework to assess the public health impact of policy change	Evaluation framework
Johansson [11]	2010	Sweden	Human services	Policy	N/A	N/A
Leeman et al. [63]	2012	USA	Obesity prevention	Policy	Center TRT’s evaluation framework	Evaluation framework
Lipsky [13]	1980	USA	Social services	Policy and individual	Street-Level Bureaucracy	Implementation theory
Matland [64]	1995	USA	Policy implementation	Policy	Ambiguity-Conflict Model of Implementation	Implementation theory
Mendel et al. [65]	2008	USA	Mental health	Organizational/community	Framework of Dissemination in Health Services Intervention Research	Process framework (2^nd^)
Michie [66]	2011	UK	Behavior change (EBPs)	Individual	The Behaviour Change Wheel	Implementation theory + determinants framework
Moulton and Sandfort [67]	2017	USA	Public service interventions	Policy	The Strategic Action Field Framework	Implementation theory
Pettigrew and Whip [68]	1992	UK	Business	Organizational/firm	Understanding strategic change: three essential dimensions (Warwick Framework)	Classic theory
Proctor et al. [69]	2011	USA	Mental health	Implementation outcomes	Conceptual Model of Implementation Research	Evaluation framework
Raghavan et al. [70]	2008	USA	Mental health	Policy	A Policy Ecology of Implementation	Determinants framework
Rutten et al. [71]	2003	Germany/Europe	Health promotion	Policy	Determinants of policy analysis	Determinants framework + classic theory
Sabatier and Mazmanian [14]	1980	USA	Policy implementation	Policy	Framework of Analysis for the Implementation of Public Policy	Determinants framework + process model
Schoenwald et al. [72]^a^	2008	USA	Mental health	System	Conceptual model for the MacArthur research network on youth mental health child STEPs initiative on evidence-based practice in clinics and systems	Determinants framework
Shortell [73]	2004	USA	Health care	System	N/A; levels and associated assumptions about change	Implementation theory
Spoth et al. [74]	2013	USA	Public health/prevention	Population	Translation Science to Population Impact (TSci Impact) framework	Process model
Strehlenert et al. [75]	2015	Sweden	Health and social care	Policy	Conceptual Model for Evidence-Informed Policy Formulation and Implementation	Process model
Thomann et al. [76]	2017	Germany	Policy implementation	Policy	Extended Accountability Regimes Framework	Implementation theory
VanDeusen Lukas et al. [77]	2007	USA	Heath care	Organizational	Framework for Organizational Transformation	Classic theory
Viennet and Pont [78]	2017	International	Education	Policy	Education Policy Implementation Framework	Determinants framework and implementation theory
Wandersman et al. [79]	2016	USA	Empowerment evaluation	Innovation and system interface	Getting to Outcomes	Process model
Wisdom et al. [80]	2014	USA	Innovation adoption	System	N/A	Determinants framework

^a^Also included in empirical literature

**Table 3:** Overview of Included Empirical Literature

Author	Year	Author location	Topic area	Level of focus	Methodology
Bax et al. [81]	2010	Netherlands	Road safety	System	Policy analysis
Beidas et al. [82]	2016	USA	Mental health	Stakeholder	Qualitative interviews
Brodowski et al. [83]	2013	USA	Child abuse prevention	System	Descriptive case study
Brownson et al. [84]	2015	USA	Public health	System (state + local)	Cross-sectional survey
Chaudoir et al. [9]^a^	2013	USA	Health	System (measures of determinants)	Systematic review and criterion-validity assessment
Cheadle et al. [85]	2009	USA	Physical activity promotion	Community	Evaluation—uncontrolled prospective design
Culotta et al. [86]	2016	USA	Climate change	Regional	Case study/policy analysis
Evans [87]	2013	UK	Health	Policy	Mixed methods survey and in-depth interviews
Fleuren et al. [88]	2014	Netherlands	Prevention child health care/schools	Innovation determinants	Systematic review + Delphi study
Gotham et al. [89]	2008	USA	Addictions	System (state)	Case study
Grace et al. [90]	2015	Australia	Mental health	Policy	Policy analysis (document analysis)
Grundy and Smith [91]	2011	Canada	Employment	Policy	Policy analysis
Hargreaves et al. [92]	2013	USA	Home visiting	Systems	Mixed methods
Haug et al. [93]	2010	Europe	Climate change	Policy	Literature review
Horner et al. [94]	2014	USA	School behavioral supports	Multi-state EIPP	Descriptive evaluation
Monroe-DeVita et al. [95]	2012	USA	Mental health	EIPP	Literature Review
Painter [96]	2010	USA	Mental health	Policy	Single case study (document analysis + secondary data analysis of single provider)
Perla et al. [97]	2013	US and UK	Healthcare	System	Scan of literature using modified Delphi technique
Powell et al. [98]	2012	USA	Health and mental health	EIPP	Narrative review
Powell et al. [99]	2014	USA	Mental health	EIPP	Systematic review
Powell et al. [100]	2015	USA	Health and mental health	EIPP	Delphi process
Rhoades et al. [101]	2012	USA	Prevention (of crime and delinquency)	System (state level)	Case description
Rieckmann [102]	2011	USA	Addictions	Policy	Mixed methods (survey and key informant interviews)
Rieckmann [103]	2015	USA	Addictions	Policy	Mixed methods (survey and key informant interviews)
Rubin [104]	2016	USA	Alignment of implementation and public systems	Systems	Intro to special issue (review of articles)
Schoenwald et al [72]^a^	2008	USA	Mental health	System	Structured survey (national sample)
Yamey [105]	2012	USA	Health in LMICs	System	Key informant interviews
Zhang and Marsh [106]	2016	China	Administrative policy transfer	Policy	Policy analysis
National Implementing Evidence-Based Practices Project articles (53 sites; 8 states), *n* = 9^b^					
Bond et al. [27]	2009	USA	Mental health	System (multi-state)	Mixed methods
Finnerty et al. [28]	2009	USA	Mental health	Policy/system	Instrument development and testing
Isett et al. [29]	2007	USA	Mental health	System (multi-state)	Qualitative (interviews)
Isett et al. [30]	2008	USA	Mental health	System (multi-state)	Case study (site visits + semi-structured interviews)
Jones et al. [31]	2014	USA	Mental health	System (multi-state)	Semi-structured interviews (state leaders)
Mancini et al. [32]	2009	USA	Mental health	Innovation (2 states)	Mixed methods (fidelity measurement + interviews, surveys, and site visits)
Peterson et al. [33]	2014	USA	Mental health	System (multi-state)	Longitudinal analysis (descriptive)
Rapp et al. [34]	2005	USA	Mental health	System (multi-state)	Descriptive
Rapp et al. [35]	2010	USA	Mental health	System (state)	Descriptive

^a^Also included in conceptual

^b^Nine articles described individually in subsequent rows

### General observations

Through this process, we noted several general observations regarding the characteristics of existing literature. In terms of the scholarly disciplines, most of the implementation science literature focused on the organizational or service provider levels with an emphasis on changing practice, often by introducing an evidence-informed policy or practice (EIPP). The knowledge translation literature included policymakers as a target audience for research evidence, but the focus was on the agenda setting or policy formulation stages of the policy cycle, as opposed to the implementation of an EIPP. Here, the scholarship focused on strategies to increase the use of evidence in policy decision-making. The public policy literature included theory describing “top-down”, “bottom-up”, and integrated approaches to implementing an EIPP. The object of implementation in this area was the policy itself, rather than a specific program or practice. There was often no clear articulation of independent and dependent policy-related implementation variables across any field, although many articles did partially address this.

### How policy is described in implementation theory and processes

Our coding based on the compass question resulted in the following characterization of how policy is described in implementation theory and processes:

Policy is described as follows:

1. Context in which implementation occurs (i.e., only briefly citing a policy as the reason for implementation)
2. Focusing lens, signaling to systems what the priorities should be (i.e., referring to policy statements or attention by policymakers as a signal about what is important to prioritize)
3. Innovation itself—the implementation object (i.e., the “thing” being implemented is policy, such as new legislative policy on tobacco cessation)
4. Lever of influence in the implementation process (i.e., policy is identified as at least one of the factors influencing the implementation process)
5. Enabler/facilitator or barrier to implementation (moderating variable) (i.e., while policy is identified as being external to the implementation effort, it is later found to be a barrier or facilitator to implementation)
6. Outcome—the success of the implementation process is at least partially defined and measured by a change in policy.
7. Policy actors as important participants or leaders in implementation

### Theoretical framework

Our approach to developing the theoretical framework was twofold. The findings from our analysis suggested constructs that addressed both the process of implementation and the factors underpinning the success or failure of implementation. We therefore first developed a process model [3] that describes the steps in the process of translating EIPPs into effectively embedded system changes. Next, we developed a determinants framework, which specifies the types of policy determinants (independent variables) that affect implementation outcomes (dependent variables). This two-part theoretical framework achieves two goals: (1) the process model is most useful in describing the process of implementation from a policy perspective and (2) the determinants framework is most useful for understanding and explaining policy-related influences on implementation outcomes.

### Part 1—process model

**Fig. 2:**
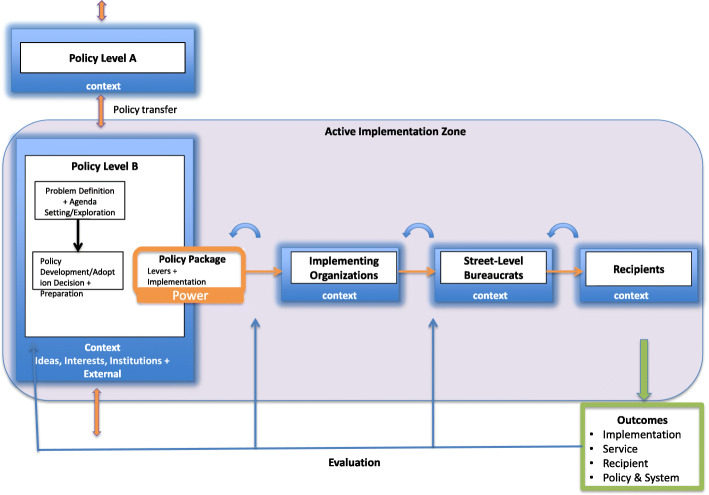
Process model of implementation from a policy perspective depicting the process at one policy level

Figure 2 depicts this novel process model focusing on one policy or system level. What follows is a narrative description of the model.

Policy is shaped as it moves through systems. The process through which policy travels from one level to another is known as policy transfer [36, 45, 46]. Each policy level is nested in a context that includes existing ideas (values, evidence, etc.), interests (interest groups, civil society, etc.), institutions (existing rules and institutional structures), and external factors (natural disaster, change in economic conditions) that affect the interpretation of the policy package [107, 108]. This context affects how a problem is defined, whether it has the attention of decision makers and whether it is up for active decision-making. This aligns with the “problem definition” and “agenda setting” stages of the policy cycle but is also described as part of the “exploration phase” in implementation science [12, 109]. Once a decision has been reached that something should be done to address a given issue, attention shifts to the “policy development” stage of the policy cycle, which aligns with the “adoption decision and preparation” stage of implementation. It is during the policy development/adoption decision and preparation stage that the policy package gets developed.

#### Policy package

A policy package usually includes a mix of policy levers or instruments, including legal and regulatory instruments, economic instruments, voluntary instruments, or information and education instruments [58, 110]. The policy package can also include some implementation guidance such as a description of the overall implementation strategy architecture, the major streams of activity, timing of events and milestones, and roles and responsibilities.

The level of ambiguity of the policy package in terms of its goals and means of attaining them, and the amount of conflict among actors with respect to the policy package are important to help characterize the implementation process and to explain its outcomes. According to Matland [64] the consideration of ambiguity and conflict leads to four types of implementation processes: (1) administrative implementation occurs when there is low policy ambiguity and low policy conflict (e.g., eradication of small pox), (2) political implementation occurs when there is low ambiguity but high levels of conflict (e.g., public transit), (3) experimental implementation occurs when there is high ambiguity but low conflict (e.g., Head Start programs for young children), and (4) symbolic implementation occurs when both ambiguity and conflict are high and policies only have a referential goal and differing perspectives on how to translate the abstract goal into instrumental actions (e.g., establishing youth employment agencies).

#### Implementation process

The policy implementation process can start at any level, move in any direction and can “skip” levels. Power also shifts as implementation proceeds through levels [29, 56]. The level with the most implementation activity tends to have the most power. This is true not only for different levels of governance, but as implementation cascades across organizations, through “street level bureaucrats” [13] and on to the end-user or target population (the “recipient”) of the implementation process. Policy decisions at one level become context for other levels. Implementation activities at one level can exert either direct or indirect effects on another level. The context surrounding each level (prevailing ideas, interests, institutions, and external events) influences the acceptability and ultimate success of implementation. Finally, the overall implementation approach may need to shift over time in response to a constantly evolving context. For example, one study found it necessary to change the implementation approach for a road safety program in respond to changes in policy authority [81].

#### Outcomes

The process of implementation is undertaken in order to lead to outcomes, which can be separated and measured at different levels. Proctor et al. [69]e identifies three separate outcomes: (1) implementation outcomes, (2) service outcomes, and (3) recipient-related outcomes. Along with these outcomes, our model includes policy- and systems-level outcomes. These can be evaluated according to the policy outputs (i.e., enforcement variables, change of perspective of street-level staff), policy outcomes (i.e., unemployment levels, life-expectancy of population) or indices of policy system change (i.e., administrative re-organization, privatization) [56]. While the measures and levels will vary depending on the size, scale, and focus of implementation, there is broad agreement that outcomes should be clearly defined a priori and precisely measured. Evaluation findings regarding outputs and outcomes can dynamically feed back into the implementation process as it unfolds. This creates feedback loops and the process becomes very dynamic and multi-directional.

### Part 2—determinants framework

**Fig. 3:**
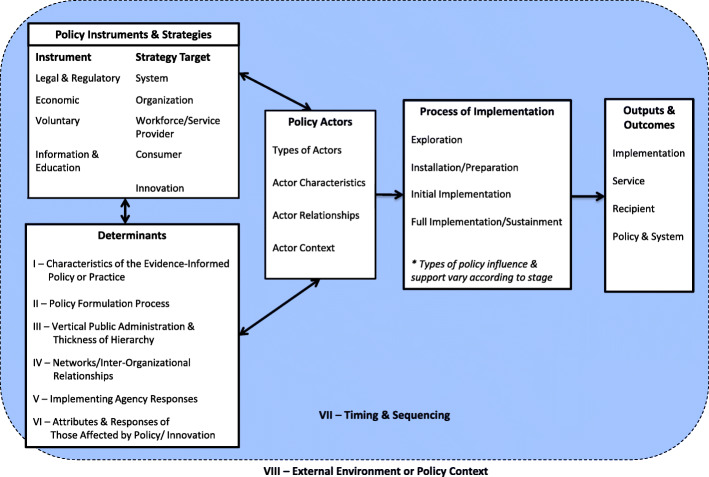
Determinants framework of implementation from a policy perspective

Figure 3 presents an overview of our determinants framework and the relationship among the determinants. Our findings point to three sets of policy-related factors that affect the process, outputs, and outcomes of implementation: (1) policy instruments and strategies, (2) determinants of implementation, and (3) policy actors, including their characteristics, relationships, and context. Collectively, these feed into the process of implementation that proceeds in an iterative fashion along the stages: exploration, installation/preparation, initial implementation, and full implementation/sustainment [12, 109]. The types of policy influences vary according to the stage of implementation [12]. The process of implementation leads to a variety of outputs and outcomes as described above.

#### Policy instruments and strategies

Policy instruments and strategies are the most common set of factors mentioned in the literature and we found evidence for each of the instrument types described here, although with varying levels of detail. Policy instruments can be applied to implementation in differing ways, often with two or three levers used concurrently to implement a single initiative or strategy [90]. In order to classify these strategies in a meaningful way, we drew on and adapted elements of a mutually exclusive and collectively exhaustive framework that identifies key features of health and social systems [107] and honed in on strategies that are particularly important for implementation (Table 4). These include strategies focused on the governance arrangements, financial arrangements, service delivery arrangements, and implementation-related supports in systems. We then divided these strategies according to the intended “target” of implementation. Common targets of implementation from a policy perspective include the whole system, organizations, the workforce or service providers, consumers, and the innovation itself (the EIPP to be implemented). We wish to note, however, that because policy-related variables have not necessarily been treated with the same specificity as other types of implementation variables, the most common strategies do not reflect the full array of strategies that *could* be employed.

**Table 4:** Policy-related strategies and examples of those strategies for implementation according to type of target

Target	Strategy	Examples	References
**System**	Policy authority (governance arrangement)	• Centralization/decentralization of policy authority (e.g., creating a regional infrastructure with some policy authority to oversee implementation) • Accountability of the state sector’s role in implementation (e.g., develop system-wide performance indicators or targets, monitor performance and fidelity, evaluate, report results publicly, consider enforcement strategies) • Leadership for implementation (through the appointment of state sector leaders, dedicated resources, garnering support for innovation and its implementation) • Stewardship of the non-state sector’s role in implementation (e.g., constructing formal opportunities for non-state sector in oversight of implementation, contracting with non-state sector for implementation-related activities, fostering networks and linkages across different types of organizations who are engaged in implementation)	[7, 12, 27–31, 38, 39, 45, 80, 100]
	Funding system infrastructure (financial arrangement)	• Dedicate resources for system infrastructure to support implementation (e.g., intermediaries, technical assistance centers, backbone organizations, facilitators) • Create funding sources that align with time needed for effective implementation and scaling	[9, 27, 28, 31, 32, 35, 39, 49, 74, 82, 83, 94, 97, 100, 101]
	Re-designing system to meet needs (delivery arrangement and implementation-related supports)	• Consider impacts of implementation on availability of care/service and plan for scaling-up across the geographical area or population • Assess possible impacts on other services (e.g., wait times) in response to implementing innovation	[7, 14, 26, 34, 37, 50, 53, 54, 73, 89, 96, 100]
	With what supports service is provided (delivery arrangement)	• Create or change system-wide record systems or information and communication technologies to support implementation	[29, 94, 97, 100]
**Organization**	Organizational authority (governance arrangement)	• Management approaches in support of optimal implementation, including: developing data collection systems, developing and monitoring performance indicators, quality improvement plans, use of scorecards, or public reporting • Develop and deploy appropriate organizational leadership for implementation oversight and engagement • Include innovation as part of accreditation processes • Engage in networks/multi-institutional arrangements in support of implementation	[7, 34, 72, 89, 97, 100]
	Funding organizations (financial arrangement)	• Provide service grants or contract with organizations to support implementation or to offset additional administrative costs of implementing an innovation (e.g., training, data infrastructure changes, workforce stability impacts) • Prospective payments to cover lag-time costs when beginning to implement an innovation • Targeted payments or penalties based on organizational performance related to innovation (e.g., changing reimbursement rate structure so that providers of high fidelity receive modestly higher per unit rate) • Targeted payments or penalties based on client outcomes • Shift organizational funding models to support implementation (e.g., from fee-for-service to no-risk managed care arrangements)	[12, 28, 29, 34, 35, 70, 72, 94, 96, 100, 103, 104, 111]
	Where service is provided (delivery arrangement)	• Adjust sites of service delivery in response to an innovation • Consider how the physical structure, facilities, and equipment can support innovation during implementation and ensure appropriate supply (supply chain management) • Adjustments to the organizational scale in response to an innovation (e.g., number of beds, units of service)	[90, 100]
	With what supports service is provided (delivery arrangement)	• Change organizational record systems or other information and communication technologies to support implementation	[97, 100]
	Organization-targeted implementation supports (Implementation-related supports)	• Develop educational materials, hosting educational meetings, training, or outreach visits tailored to organizations • Develop and disseminate program or organizational service standards • Provision of technical assistance and other forms of implementation support • Support development and maintenance of inter-organizational collaboratives, communities of practice, and other forms of inter-organizational communication/learning • Consider non-monetary awards, incentives, and disincentives for organizations (e.g., exemplary program award)	[7, 9, 28, 29, 35, 52, 53, 65, 82, 86, 100]
**Workforce/service provider**	Professional authority (governance arrangement)	• Create or alter training and licensure requirements • Change scope of practice to reflect innovation • Alter where providers can practice geographically and in what systems (public vs private) • Continuing competence (e.g., provide training and continuing education unit credits for innovation or disallow certain courses for credit) • Professional liability (e.g., change liability laws) • Alter university curricula to include knowledge of innovation	[27, 28, 34, 70, 72, 89, 95, 100]
	Remunerating providers (financial arrangement)	• Reimbursement for program participation, extra efforts in applying the innovation, or lost time due to training • Increase reimbursement rate • Changing the way providers are reimbursed to encourage implementation (e.g., from fee-for-service to capitation) • Loan forgiveness • Targeted payments or penalties for performance • Targeted payments or penalties based on outcomes • Review and align fiscal and billing policies and incentives for providers • Make billing easier for providers	[7, 27, 32, 34, 47, 52, 65, 69, 70, 73, 80, 82, 88, 90, 100, 104]
	By whom service is provided (delivery arrangement)	• Assess and improve workplace conditions for providers to foster implementation • Extend the role of a particular provider within their existing scope of practice • Shift tasks between types of providers • Optimize the performance of the workforce in their current roles by creating, disseminating, and monitoring guidelines or standards of care for service providers	[7, 45, 49, 54, 57, 66, 79, 89, 100]
	Workforce-targeted implementation supports (implementation-related supports)	• Develop educational materials, hosting educational meetings, training, or outreach visits • Engage local opinion leaders • Reminders and prompts • Audit and feedback • Coaching • Develop either tailored or multi-faceted interventions to support implementation • Consider non-monetary awards, incentives, and disincentives for workforce	[28–30, 34, 39, 65, 70, 83, 89, 100]
**Consumer**	Consumer and stakeholder involvement (governance arrangement)	• Consumer protection (laws, complaints management) • Consumer, family, and stakeholder engagement in implementation and monitoring	[70, 72, 89, 100]
	Incentivizing consumers (financial arrangement)	• Alter consumer/patient fees • Consider disincentives that may exist for consumers to be successful (e.g., some employment programs) • Subsidies for private health insurance	[29, 90, 100]
	Consumer-targeted implementation supports (implementation-related supports)	• Information or education provision • Behavior change support • Skills and competencies development • Communication and decision-making facilitation	[45, 54, 66, 70]
**Innovation**	Commercial authority (governance arrangement)	• Adjust licensure and registration requirements to support implementation • Consider pricing and purchasing • Establish voluntary agreements on advertising	[54, 66]
	Purchasing products and services (financial arrangement)	• Changes to the scope and nature of insurance plans: extending or ending insurance coverage • Adjust list of covered/reimbursed services and products • Change restrictions or caps on coverage/reimbursement for innovation and related supports • Change mechanisms for billing • Prior approval requirements	[32, 49, 70, 72, 79, 84, 89, 95, 100]

#### Determinants

Our framework identifies eight categories of determinants (see “Determinants” box and elsewhere in Fig. 3). Each of these categories represents a suite of factors that are hypothesized to independently affect implementation outcomes. These determinants are described briefly below and in more detail in Table 5.

**Table 5:** Determinants of implementation from a policy perspective and the factors that characterize the determinants

Determinant	Description	Factors that characterize determinant
I. Characteristics of the EIPP	1. Not possible to predict the success or failure of a particular policy package based on its intrinsic characteristics alone [56] 2. Need to examine questions such as whether the policy selected: (a) Is an appropriate fit with the problem [78, 91] (b) Aligned with existing context [12, 88]	1. Relative advantage [26, 37] 2. Compatibility [26, 37] 3. Complexity of goals and ease of implementation [26, 37, 67] 4. Obligations [26, 37] 5. Resources [26, 37] 6. Existing relationship with state and provider [103] 7. Level of ambiguity of the EIPP [64] 8. Level of conflict among stakeholders [64] 9. Interaction of policy characteristics with other determinants [56]
II. Policy formulation process	1. Shape given to a policy by the initial formation processes has an impact on its implementation [56] 2. Depending on the implementation approach, the government may distribute responsibility for some or almost all of the policy formulation process to other stakeholders [12] 3. Level of involvement of service organizations, street-level bureaucrats and recipients may influence the confidence in, and support of, the policy decision and improve the chances for successful implementation [97]	1. Government actors responsible for formulating policy [56] 2. Perceived legitimacy of government actors [56] 3. Extent to which there is opportunity to provide feedback [56] 4. Responsiveness of policymakers to feedback [56] 5. Level of involvement of non-governmental actors [12, 78, 97] 6. Adequacy of planning for implementation (consideration of resources for implementation) [97] 7. Constraints experienced during formulation [12, 26]
III. Vertical public administration and thickness of hierarchy	1. Vertical Public Administration (a) Term used to identify the layers in the policy transfer process (b) Refers to separate governments exercising their authority with relative autonomy [56] (c) Policies generated outside of a socio-political level may be more or less acceptable to that level 2. Thickness of Hierarchy (a) Number and complexity of institutions, departments, or agencies at a particular socio-political level (b) The thicker the hierarchy, the more managerial competence, professionalism, and governance skills are required by public servants in order to support effective implementation [59]	1. Number of socio-political levels [56] 2. Acceptability of policy generated outside of a particular socio-political level [114] 3. Appropriateness of socio-political level [86] 4. Thickness of each socio-political level (number and complexity of institutions, departments, or agencies and their coordination and interdependence) [55]
IV. Networks/inter-organizational relationships	1. Reflects the existence and nature of the relationships between parallel organizations who must collaborative in order to achieve effective implementation and who do not have a hierarchical relationship [56] 2. Better connections among stakeholders also increases the opportunity for rapid diffusion and informal spread of innovation, facilitating implementation	1. Degree of coordination among: (a) Systems [80] (b) Organizations [86] (c) Donors /other funders [84] (d) Leaders [12] 2. Formality (formal or informal) [72] 3. Network type (e.g., policy or inter-organizational) [53] 4. Coherence and strength of connections [112]
V. Implementing agency responses	1. Factors affecting the responses of implementing agencies can be divided into: (a) Issues related to the overall characteristics of the agencies (b) Behavior of front-line or street-level staff [56] 2. Overall “health” of organizations and how front-line/street-level bureaucrats use their discretion and power impact implementation success	1. Overall characteristics of the agencies: (a) Level of organizational control [56, 76] (b) Rate of staff turnover [88] (c) Organizational decision-making processes [88] (d) Extent of policy and behavior-related change required [14, 67] (e) Attitudes of the agencies [14, 47] (f) Resources of the agencies (e.g., minimum “investment threshold” in implementation infrastructure [97] or cost-absorptive capacity of agency to absorb additional costs associated with implementation [12] or certainty of funding [49]) (g) Impetus for chang e[77] (e.g., external mandates may increase an agency’s predisposition (i.e. motivation), but not its capacity to adopt an innovation; mandates may divert activity away from other innovations or locally generated priorities [26]) (h) Perception of implementation approach (e.g. if approach is punitive, mandatory, or “top down”) [82] 2. Behavior of front-line or street-level staff (a) Level of discretion and level of relative autonomy from organizational authority affect the amount of interpretation of EIPP [13, 76] (b) Competing accountabilities (e.g., state, market, professional, societal) [76] (c) Power distribution between actors at the front-line, agency, and political levels [30] (d) Personal characteristics including their knowledge, skills, and perceived support from colleagues [88]
VI. Attributes and responses from those affected by EIPP	1. The characteristics of the people affected by the EIPP, their response to it, and the impact of the responses 2. Most evident when those affected are powerful, such as in regulatory policy when those regulated are large organizations [56]	1. Diversity of target group behavior [14] 2. Target group as percentage of the population [14] 3. Impacts on stability of the workforce and responses to instability [12]
VII. Timing/sequencing	1. Implementation processes at scale require adequate time, which does not always align with the cycles they are subject to and some authors have identified the lack of time or short-term opportunism as barriers to effective implementation [e53, 54] 2. The sequencing of activities and alignment of implementation with other cycles is also important	1. Balance of predictability and adaptiveness to changing circumstances [68, 93] 2. The simultaneous address of system levers (including policy changes, measurement systems, and regulatory mechanisms) [97] 3. Timing and pace of cycles, such as political, policy, and funding cycles [104] 4. Specific aspects of time that impact implementation: (a) The phased structure of the implementation process [104] (b) When and how the implementation efforts are initiated [104] (c) Timeframes for funding and leadership support [104] (d) The need to demonstrate the impacts early (e) Return on investment of time and money [104]
VIII. External environment or policy context	1. Factors outside of the policy area of focus may influence implementation 2. Can be referred to generally as the “political and social climate”, as unmodifiable or macro “context” or as “socio-economic conditions” [9, 14, 38, 40, 52, 70, 75, 80] 3. While most included articles did not address these determinants in depth, an overall examination of extracted data suggested two theoretical frameworks would be useful for classifying and understanding these determinants: (a) 3I+E framework that identifies the institutions, interests, ideas, and external events that help explain what influences policy choices [113] (b) Taxonomy of health and social system arrangements classified according to the governance, financial, and delivery arrangements [114] 4. These broader context and system arrangements may be critically important in explaining implementation outcomes and these frameworks provide some logic and organization to potential variables	1. 3I+E framework (a) Ideas (e.g., the interplay between beliefs and values of policymakers and research evidence in a general way [38]) (b) Interests (e.g., the political culture and the depth of social cleavages [58]) (c) Institutions (e.g., relevant policies from other areas that “may represent potentially powerful contextual effects” [74, 78]) (d) External factors (e.g., technology and technological changes [14, 40]) economic forces operating in the overall society [60], and environmental (in)stability [53, 67] 2. Taxonomy of health and social system arrangements [114]. (a) Governance arrangements that are not specific to the EIPP being implemented but are still relevant to understanding implementation outcomes (e.g., centralization and power distribution of government [30, 72] or the form of governance structures (omnibus/discrete) [72]) (b) Financial arrangements (e.g., private/public contractual relations, reimbursement rates and mechanisms [72], and existing resource distribution [30]) (c) Delivery arrangements—referred to more generally in the health-focused articles as “health(care) system and services context” [38, 75] or “medical delivery system” [40]

*I—Characteristics of the evidence-informed policy or practice (EIPP).* The success or failure of a particular policy package cannot be evaluated based on its intrinsic characteristics alone [56]. Instead, it is important to examine whether the policy selected is an appropriate “fit” with the problem [91], well-justified [78], and aligned with existing context [12, 88].

*II*—*Policy formulation process.* This is the shape given to a policy by the initial formation processes [45]. It includes who in government is responsible for formulating the policy, their legitimacy and the extent to which there is opportunity to provide feedback, how much feedback is given, and the responsiveness in terms of adjustments made [45].

*III—Vertical public administration and thickness of hierarchy.* Vertical public administration is the term used to identify the layers in the policy transfer process. It refers to separate governments exercising their authority with relative autonomy [45]. Policies generated outside of a socio-political level may be more or less acceptable to that level. Within a given layer, a particular policy area may require the mobilization of any number of institutions, departments, or agencies, and these agencies must act in a coordinated, interdependent fashion, termed “thickness of the hierarchy” [55].

*IV—Networks/inter-organizational relationships.* The existence and nature of the relationships between parallel organizations who must collaborative in order to achieve effective implementation and who do not have a hierarchical relationship [45].

*V—Implementing agency responses.* The factors affecting the responses of implementing agencies can be divided into issues related to the overall characteristics of the agencies and the behavior of front-line or street-level staff [13, 56].

*VI—Attributes and responses from those affected by EIPP*. Attributes include the diversity of target group behavior and the target group as a percentage of the population [14]. Responses include thing like impacts on workforce stability [12].

*VII—Timing/sequencing.* As implementation is a process that unfolds over time, it does not always align with the cycles to which it is subject and the time constraints inherent therein [86, 87]. Additionally, the external context in which implementation occurs is ever changing and “quintessentially unstable”, and success hinges on the ability to perceive those changes and take the necessary actions to adjust along the way [68]. In Fig. 3, timing/sequencing is placed outside of the determinants box to reflect its importance across all of the other elements.

*VIII—External environment or policy context.* Much of the literature identified factors outside the policy area of focus that may influence implementation (Fig. 3, outside the hatched line). Many authors referred to this generally as the “political and social climate”, as unmodifiable or macro “context”, or as “socio-economic conditions” [9, 14, 38, 40, 52, 70, 75, 80]. We organized this determinant using (1) the 3I+E framework [113] and (2) a taxonomy of health and social system arrangements [114].

In general, these categories of determinants should be viewed as interactive and not completely discrete [56] and the inter-relationship among the determinants is key [45].

#### Policy actors

Our analysis revealed a wide range of policy actors who are important for implementation. In an attempt to create a category of variables that is analytically useful across contexts, we first divided the types of policy actors into the broad categories of political actors, bureaucratic actors, special interests, and experts [115]. To provide more specificity, we further divided these into a non-exhaustive list of actor sub-types that were frequently mentioned in the literature and included examples of the types of roles they tend to assume in implementation (Table 6). While many of the sub-types are commonly identified in other phases of the policy cycle, some receive particular attention in the implementation literature. These include two types of special interests: (1) implementing agencies, organizations or programs that are responsible for implementing the EIPP (e.g., hospitals, schools), and 2 street-level bureaucrats who, due to the relatively high degree of discretion in their jobs, and therefore discretion over the dispensation of public benefits or sanctions to citizens, can be critical to realizing any large-scale implementation efforts. There are also three expert sub-types that are particularly visible during implementation: (1) field or practice leaders who can be influential in supporting practice change among professionals, (2) innovation developers/disseminators who have developed the EIPP to be implemented and who may contribute or adapt tools and other types of support to encourage successful implementation, and (3) intermediaries/technical assistance providers who are organizations, programs, or individuals that work “in between” policymakers, funders, and front-line implementers, to facilitate effective implementation drawing on expertise in implementation.

**Table 6:** Types of policy actors identified in implementation

Actor	Sub-type (non-exhaustive)	Role description	Role in implementation (non-exhaustive)	References
**Political actors**	Politicians	• Represent citizens (in a democracy) through popular consensus. • Mandate to create laws and policies with varying levels of authority • Can be supra-national, national/federal, state/provincial, regional, local/municipal	• Most important level of elected officials is the level where most policy authority rests for area of implementation • Develop and pass laws/policies (e.g., mandating a particular EIPP) • Provide leadership and focus • Source of funding for implementation (organizations, providers, and/or consumer levels)	[34, 36, 41, 50, 54, 61, 63, 65, 80, 82, 83, 94, 95, 101]
	Other elected officials	• Similar to elected politicians but mandate is limited to a particular policy domain and (often) limited geographic jurisdiction (e.g., sheriff, judge, school board trustee)	• If policy authority rests at their level, they may develop and pass laws • Enforce laws/polices from other levels • Interpret/adapt laws/policies for their implementation • Provide leadership and focus • Source of funding for implementation (organizations, providers, and/or consumer levels)	[54]
**Bureaucratic actors**	Executive departments	• Departments or ministries who specialize in a unique area of government responsibility (e.g., health) • Responsible for carrying out the “vision” of an elected official with leadership for that portfolio (e.g., Minister of Health) • Not elected nor formally tied to a particular political party	• Support policy development, including implementation considerations • Operationalize policy/law passed by politicians • May allocate tasks, responsibilities and define competencies for implementation • Monitor policy implementation and track outputs or outcomes • Source of funding for implementation (organizations, providers, and/or consumer levels)	[34, 61, 82, 84, 89]
	Boards and agencies of government	• Often operate semi-independently from government but are appointed by them • In most cases, they deal exclusively with one particular sub-field of responsibility in which the demand for public services is especially high (e.g., food inspection agency, state mental health authority)	• Regulation and enforcement • Interpretation of policies/laws • Monitor policy implementation and track outputs or outcomes • May have the ability to apply penalties for non-compliance • May allocate tasks, responsibilities, and define competencies for implementation	[27, 28, 34, 65, 80, 82]
	Self-governing regulatory agencies	• Bodies that regulate the conduct of their own members (such as admissions and discipline) and are empowered to do so by the appropriate level of government and their members (e.g., medicine, law) • Regulators are drawn from the membership	• Can set or change: scope of practice, training, and licensure requirements, or professional liability to support implementation • Develop/adopt guidelines or standards • Monitor quality and safety and continued competence of professionals during implementation	[82]
	Judicial system	• System of courts that provide a formal mechanism for interpretation and application of laws in the name of the state and resolves disputes	• Interpret/re-interpret laws through rulings that may affect how they are implemented • Define/re-define public policies through legal challenges	[40]
**Special interests**	Implementing agencies	• Organizations or programs that are responsible for implementing the laws or policies developed (e.g., hospitals, schools, child welfare agencies, industry) • Location(s) where the majority of the implementation takes place	• Interpretation of policies/laws • Develop or adapt organizational policies and procedures to support implementation • Training and support for workforce • Provide or manage funds to support implementation • Monitor and evaluate implementation at organizational level	[28, 34, 41, 43, 50, 83, 87, 89, 91, 95, 101]
	Street-level bureaucrats	• The schools, police and welfare departments, lower courts, legal services offices, and other agencies whose workers interact with and have wide discretion over the dispensation of benefits or the allocation of public sanctions [13] • Have (1) relatively high degree of discretion and (2) relative autonomy from organizational authority [13]	• Interpretation of policies/laws • Often the parties responsible for changing their behaviors or practices during implementation	[13, 41, 43]
	Insurers	• Organizations or government bodies that manage risk by pooling risk across a group of individuals and providing coverage to them for needed services • Managed care organizations are a specific type of insurer in health care that monitor and control the provision of care in an effort to increase quality through regulating the choices of providers and patients	• Have the ability to change the risk pool by insuring more or fewer people (scope and nature of insurance plan) • Can adjust the list of covered/reimbursed organizations, providers, services, and products • Can change billing/reimbursement processes to facilitate implementation • Engagement and potential influence with political and bureaucratic actors (feedback loops) regarding implementation and scaling	[50, 65, 82]
	Donors/foundations	• Organizations that raise and allocate funds based on a specific mandate that they identify	• Funding and/or in-kind implementation supports (e.g., human resources) • May have funded an innovation and now have a vested interest in seeing it implemented or scaled (bring leadership and focus, implementation, and scaling expertise) • Engagement and potential influence with political and bureaucratic actors (feedback loops) to support implementation and scaling	[41, 105]
	Government corporations	• Organizations or businesses that are run independently from government but are still ultimately accountable to them	• Interpretation of policies/laws • Develop or adapt organizational policies and procedures to support implementation	
	Unions	• Organized associations of workers created to promote and protect their interests in the workplace	• Negotiate contractual relationships with implementing organizations on behalf of members (can influence the ease of implementation) • Engagement and potential influence with political and bureaucratic actors (feedback loops) regarding implementation and scaling	[34, 41]
**Experts**	Scientists/researchers	• Individuals or research programs that systematically gather, analyze, and use research and other evidence through processes, such as theorizing, synthesizing, and hypothesis testing, to gain and share understanding and knowledge	• Share or contribute research expertise concerning the problem, the innovation, the implementation or the evaluation of the implementation effort and any expected outcomes • Engagement and potential influence with political and bureaucratic actors (feedback loops) to support implementation and scaling	[35, 101]
	Field or practice leaders/champions	• Individuals who belong to a service providing community and are viewed as leaders or champions of an innovation and its implementation	• Share or contribute practice expertise concerning the problem, the innovation, the implementation or the evaluation of the implementation effort and any expected outcomes • Act as champions for implementation to members of their service providing community and to other policy actors • Engagement and potential influence with political and bureaucratic actors (feedback loops) to support implementation and scaling	[31, 38, 42]
	Patients or persons with lived experience and families/carers	• Individuals who bring personal knowledge or experience of a problem, condition, or service and who are the intended beneficiaries or ultimate “targets” of implementation • Individuals who are family members or carers to individuals who bring personal knowledge or experience of a problem, condition, or service	• Share or contribute lived experience of the problem, the innovation, the implementation or the evaluation of the implementation effort and any expected outcomes	[28, 35, 43, 45, 57, 83, 100]
	Innovation/developers and disseminators	• Organizations, programs or individuals who have developed a process, program, or product to be implemented	• Synthesize knowledge about innovation and package it in ways that are “usable” • Actively seek opportunities for innovation to be adopted in policy and/or practice • Provide expertise about the innovation during implementation process • Adapt innovation and materials as needed during implementation process	[52, 101]
	Intermediaries and technical assistance providers	• Organizations, programs, or individuals that work “in between” policymakers, funders, and front-line implementers, to facilitate effective implementation drawing on expertise in implementation • Also known as purveyor organizations, backbone organizations, or central bodies charged with coordination	• Translate policy intention for implementing agencies • Provide technical assistance to implementing agencies (e.g., guidance on implementation process, coaching, decision support, monitoring, and evaluation) • Provide mechanism for communication between service delivery, policy systems, and innovation developer (if applicable)	[9, 31, 42, 44, 45, 53, 83, 86, 89, 100, 101]
**Other**	Media	• Individuals and organizations that communicate information through a variety of channels, including formal media outlets and social media outlets	• Monitor implementation and communicate facts or perceptions of the process and outcomes to the public • Provides feedback loop for political actors, bureaucratic actors, special interests, and experts regarding implementation	[34, 65]

**Fig. 4:**
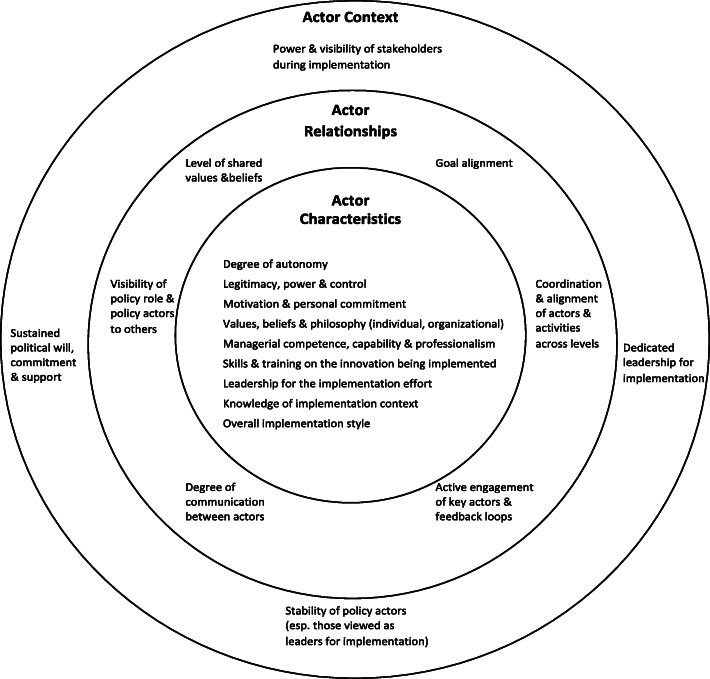
Characteristics, relationships, and the context of policy actors important for implementation

There are also three categories of actor-related variables that are important: (1) actor characteristics, (2) actor relationships, and (3) the context in which the actors are embedded (Fig. 4). First, the characteristics of the policy actors (either individual- or organizational-level) such as their knowledge of the implementation context, their legitimacy, power and control, and their leadership in the context of the implementation effort are often cited as being critical to the success in large-scale implementation initiatives. Second, the relationships policy actors have with other actors, such as the level of shared values and beliefs or the coordination and alignment of actors and their activities, can be predictive of successful implementation. Finally, the context of the actors, such as the sustainment of political will and commitment and the stability of the actors themselves can predict the long-term success of implementation.

## Discussion

Our study represents one of the first comprehensive attempts to answer the call of scholars to integrate the fields of implementation science, knowledge translation, and policy implementation in an effort to build a more comprehensive and accurate understanding of implementation. By integrating conceptual and empirical works from all three fields, the resultant two-part theoretical framework provides additional clarity regarding the process of implementation viewed from a policy perspective and identifies a number of policy-related determinants that can be tested empirically in the future.

A key strength of our study was the methodological approach we took to theory building.

First was the comprehensiveness of the search strategy, which aimed to identify scholarship from more than one academic discipline and across wide range of topics beyond health. The literature identified through the search process revealed some interesting parallels and unique differences between the fields that made it clear to us the extent of the lack of integration up to this point. Perhaps not surprisingly, the area of public health seemed to be the most fertile ground for integration. This is likely due to their focus on population-level concerns requiring system-wide implementation of EIPPs and a diverse implementation ecosystem. The search strategy was part of the mixed methods approach of the CIS, which blended the rigor of a systematic search methodology that is explicit and replicable, with the inductive, iterative, and purposive sampling techniques from qualitative review methods to build mid-range theory. The result is a theoretical framework that is clearly linked to the literature, which should instill some confidence in the academic community regarding its grounding. Critical interpretive synthesis is a relatively new approach but is growing in popularity for these reasons.

Despite the merits of our approach, we did identify some challenges. First, we believe the literature from public policy may be underrepresented for several reasons: (1) search terms did not retrieve as much from those fields (it could be that there are terms used more commonly in those fields that would have increased yield), (2) the disciplinary approach to the scholarship in public policy often means the articles were less explicit about methods and this meant that more were excluded as not being “high yield”, and (3) more of that scholarship is captured through other media (e.g., books) and while some of these were included, our approach was not as sensitive to retrieving these types of documents. We also did not include all of the empirical articles for data extraction and we may have missed a key theme or framework component. While we believe this is unlikely because we continued to sample until saturation was reached, it is still possible something was missed. Finally, there were few documents from low- and middle-income countries included in the final sample. Specific efforts to include relevant documents from LMICs in future may enrich and refine the model.

As a result of this research, policymakers and practitioners looking to use a conceptual model to guide their implementation activities have two additional options that they can be confident draw from existing theory and empirical works. Large-scale implementation endeavors or those that have started small and are looking to scale-up should at least be mindful of the critical roles of policy during the process and what policy-related factors may be important for success. Those planning implementation activities can consider the elements presented in the framework as factors that may require consideration and adjustment prior to implementing something new. Our work supports thinking beyond the program or practice levels and unpacks policy considerations that may have influence on, or affect the effectiveness of, a program or practice. Furthermore, the inclusion of policy-related outputs and outcomes in our framework offers policymakers and practitioners the option of additional indicators of success on which they can measure and report.

Like any new theoretical contribution, our framework would benefit from further refinement and testing by the research community. Future research could adopt the process model to guide a policy-intensive implementation effort and test it to determine its usefulness in such efforts. Researchers could also select particular framework elements and unpack them further for additional precision and clarity, drawing from multiple fields of scholarship. Our framework also offers some much-needed policy variables that have been lacking in the implementation science and knowledge translation fields, which could be incorporated as part of a suite of variables in implementation research.

## Conclusions

Our study represents an early effort at integrating the fields of public policy, implementation science, and knowledge translation. We have learned that there is indeed a great deal that each of the fields can learn from the other to advance our understanding of policy- and systems-level implementation efforts and hope that these efforts are followed by more interdisciplinary research in order to truly bridge this divide.

## Data Availability

Not applicable
